# Effects of Phenolic Acids Produced from Food-Derived Flavonoids and Amino Acids by the Gut Microbiota on Health and Disease

**DOI:** 10.3390/molecules29215102

**Published:** 2024-10-29

**Authors:** Yoshimitsu Kiriyama, Hiroshi Tokumaru, Hisayo Sadamoto, Suguru Kobayashi, Hiromi Nochi

**Affiliations:** 1 Kagawa School of Pharmaceutical Sciences, Tokushima Bunri University, Shido 1314-1, Sanuki 769-2193, Kagawa, Japansadamotoh@kph.bunri-u.ac.jp (H.S.); kobayashis@kph.bunri-u.ac.jp (S.K.); nochi@kph.bunri-u.ac.jp (H.N.); 2 Institute of Neuroscience, Tokushima Bunri University, Shido 1314-1, Sanuki 769-2193, Kagawa, Japan

**Keywords:** polyphenols, flavonoids, phenolic acids, gallic acid, protocatechuic acid, SARS-CoV-2, influenza, gut microbiota, gut microbiome

## Abstract

The gut microbiota metabolizes flavonoids, amino acids, dietary fiber, and other components of foods to produce a variety of gut microbiota-derived metabolites. Flavonoids are the largest group of polyphenols, and approximately 7000 flavonoids have been identified. A variety of phenolic acids are produced from flavonoids and amino acids through metabolic processes by the gut microbiota. Furthermore, these phenolic acids are easily absorbed. Phenolic acids generally represent phenolic compounds with one carboxylic acid group. Gut microbiota-derived phenolic acids have antiviral effects against several viruses, such as SARS-CoV-2 and influenza. Furthermore, phenolic acids influence the immune system by inhibiting the secretion of proinflammatory cytokines, such as interleukin-1β and tumor necrosis factor-α. In the nervous systems, phenolic acids may have protective effects against neurodegenerative diseases, such as Alzheimer’s and Parkinson’s diseases. Moreover, phenolic acids can improve levels of blood glucose, cholesterols, and triglycerides. Phenolic acids also improve cardiovascular functions, such as blood pressure and atherosclerotic lesions. This review focuses on the current knowledge of the effects of phenolic acids produced from food-derived flavonoids and amino acids by the gut microbiota on health and disease.

## 1. Introduction

A wide variety of bacteria inhabit the gut of the host and constitute the gut microbiota. The host and the bacteria in the gut microbiota exhibit a symbiotic relationship. The gut microbiota metabolizes flavonoids, amino acids, dietary fiber, and other components of foods ingested from outside the body as well as bile acids present in the body to produce a variety of gut microbiota-derived metabolites. These gut microbiota-derived metabolites affect health and disease in the host [1,2,3]. Most of the food consumed by humans is of plant origin, and those plants contain special metabolites (also called secondary metabolites). Although these specialized metabolites are not considered to function in reproduction or development in plants, they are considered to play diverse and important roles, such as a defense against disease and pest infection and alleviation of stresses such as intense light and drought [4,5,6,7]. Polyphenols are secondary metabolites produced by plants and found in a wide variety of vegetables, fruits, and grains. Therefore, humans consume polyphenols from a wide variety of foods, including beverages such as tea, wine, juice, and processed foods. Flavonoids are the largest group of polyphenols, with approximately 7000 flavonoids having been identified. Flavonoids impact human health and reduce risk factors for various diseases such as obesity, cancer, heart disease, neurodegenerative diseases, infections, and osteoporosis [8,9,10,11,12,13,14,15,16]. In addition, flavonoids, such as quercetin, corylin, and 4,4′-dimethoxychalcone were reported to decelerate senescence or reduce senescent human cells, such as human umbilical vein endothelial, osteosarcoma, cervical carcinoma, and neuroblastoma cells [17,18,19,20,21].

### 1.1. Polyphenols and Flavonoids

**Figure 1:**
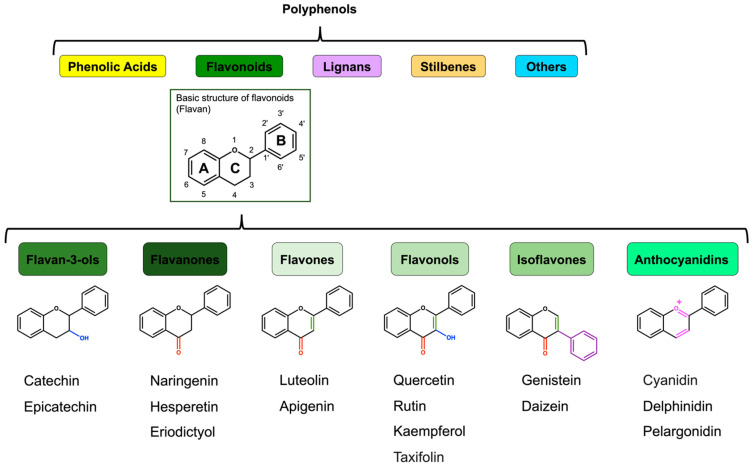
Classification of polyphenols and flavonoids. Polyphenols are classified into five classes, viz., phenolic acids, flavonoids, lignans, stilbenes, and other nonflavonoids. Flavonoids are divided into six major subclasses, including flavan-3-ols (3-OH-flavanones), flavanones, flavones, flavonols, isoflavones, and anthocyanins. The basic structure of flavonoids is a diphenylpropane skeleton (C6–C3–C6, flavan). Flavan-3-ols have a hydroxyl group at C3 in the C ring of the basic flavonoid skeleton structure. Flavanones have a ketone structure at C4 in the C ring. Flavones have a double bond at C2–C3 and a ketone structure at C4 in the C ring. Flavonols have a double bond at C2–C3, a hydroxyl group at C3, and a ketone structure at C4 in the C ring. Isoflavones have a double bond at C2–C3 and a ketone structure at C4 in the C ring, and the B ring is bound at C3 in the C ring. Anthocyanidins have double bonds at C1–C2 and C3–C4 in the C ring.

Polyphenols possess multiple phenolic hydroxyl groups, of which approximately 8000 were reported [22,23]. For humans, polyphenols provide sensory stimulation, including effects on the sense of taste, such as bitterness, and visual perception due to their unique pigments. Polyphenols are classified into polymeric and monomeric forms. The polymeric forms consist of hydrolyzed tannins, such as gallotannins and ellagitannins, and condensed tannins, such as proanthocyanidins. In contrast, monomeric forms of polyphenols are classified into five classes based on their chemical structures: phenolic acids, flavonoids, lignans, stilbenes, and others [24,25] (Figure 1). Most plant-derived phenolic acids have C1–C6 or C3–C6 in their backbones and contain benzoic acid and cinnamic acid derivatives. However, phenolic acids, which have only one phenolic group in the molecule, are phenolic compounds but not polyphenols [23,26,27,28]. In contrast, the basic structure of flavonoids is a diphenylpropane skeleton (C6–C3–C6, flavan), namely, two phenyl rings (A and B rings) and a heterocyclic C ring in their backbones [29]. Flavonoids are divided into the following six major subclasses: flavan-3-ols (3-OH-flavanones), flavanones, flavones, flavonols, isoflavones, and anthocyanins [10,30,31,32] (Figure 1). Flavan-3-ols include catechin and epicatechin. Flavanones include naringenin, hesperetin, and eriodictyol. Flavones include apigenin and luteolin. Flavonols are the most common flavonoids found in foods, including quercetin, rutin (quercetin 3-O-rutinoside), kaempferol, taxifolin, isorhamnetin, and narcissin (isorhamnetin-3-O-rutinoside). Isoflavones include genistein and daidzein. Anthocyanidins include cyanidin, delphinidin, and pelargonidin.

The average total flavonoid intake for American adults was 251 mg/day. In addition, regarding flavonoid classes, flavan-3-ol was the most abundant, accounting for 81% of the total flavonoid intake, followed by flavonols (8%), flavanones (5%), anthocyanidins (5%), isoflavones (1%), and flavones (<1%) [33].

### 1.2. Phenolic Acids from Flavonoids and Tyrosine

Most flavonoids in plants exist as glycosides (O-glycosides) in which various sugars are O-glycosidically linked to hydroxyl groups in their structures. Most flavonoid glycosides are rarely absorbed intact. However, the gut microbiota produces aglycons from various flavonoid glycosides. Moreover, the gut microbiota performs catabolic reactions such as cleavage and reduction reactions that cause structural changes in the basic skeleton of flavonoids, producing various phenolic acids from flavonoids. In addition, phenolic acids, such as 3-(4-hydroxyphenyl)propanoic acid (also known as 3-(4-hydroxyphenyl) propanoic acid or desaminotyrosine), are also produced from tyrosine (2-amino-3-(4-hydroxyphenyl)propanoic acid) through metabolic processes by the gut microbiota.

This review focused on the current knowledge of phenolic acid production by gut microbiota and its role in health and disease.

## 2. Generation of Phenolic Acids by Gut Microbiota

Phenolic acids are one of the major classes of plant phenolic molecules. The following phenolic acids are found in plants: 3-(4-hydroxyphenyl)-2-propenoic acid (also known as 4-hydroxycinnamic acid or *p*-coumaric acid), 3-(3,4-dihydroxyphenyl)-2-propenoic acid (also called as 3,4-dihydroxycinnamic acid or caffeic acid), 3-(4-hydroxy-3-methoxyphenyl)-2-propenoic acid (also known as 4-hydroxy-3-methoxycinnamic acid or ferulic acid), and benzoic acids, such as 3,4,5-trihydroxybenzoic acid, 3,4-dihydroxybenzoic acid (also known as protocatechuic acid), 4-hydroxy-3-methoxy benzoic acid (also known as vanillic acid), and 4-hydroxy-3,5-dimethoxybenzoic acid (also known as syringic acid) [26,27]. Moreover, plant-derived phenolic acids are generally present in bonded forms, such as amides, esters, and glycosides [26].

**Figure 2:**
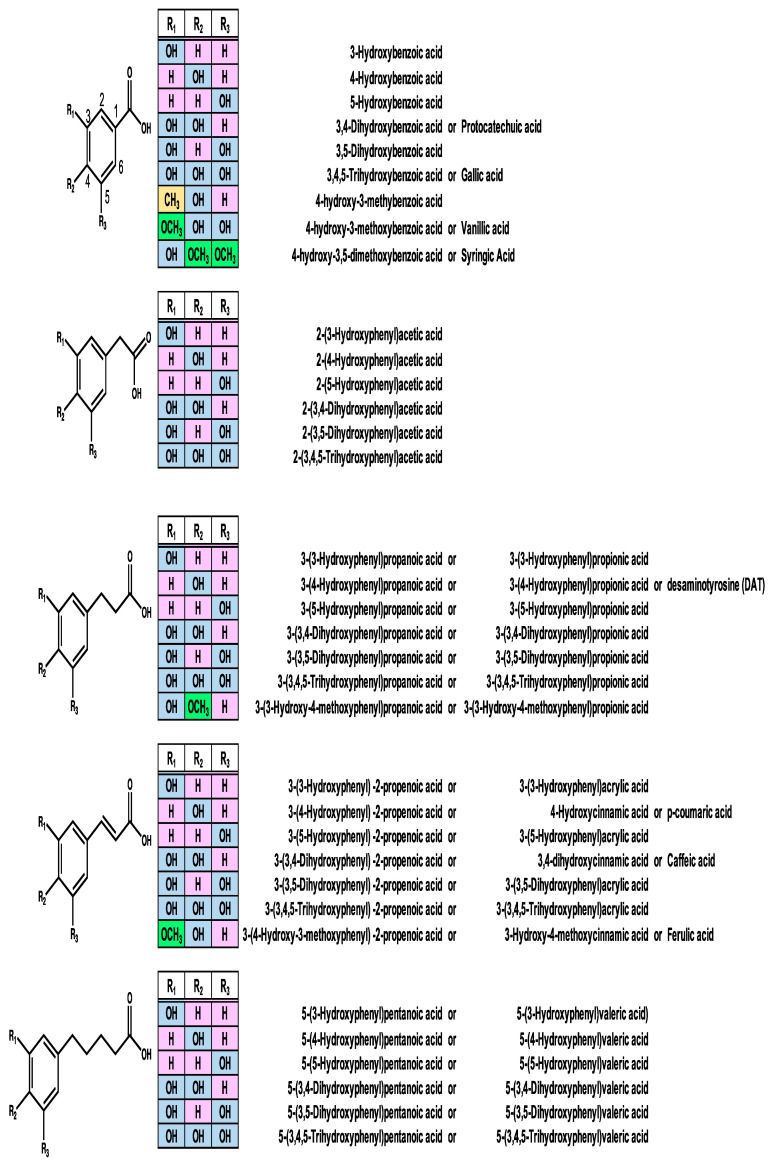
Structures of gut microbiota-derived phenolic acids.

However, phenolic acids are also produced from flavonoids and tyrosine by the gut microbiota, and the carboxylic acid groups of these phenolic acids are carboxylic acid, acetic acid, propanoic acid, and pentanoic acid (valeric acid) (Figure 2). Moreover, these phenolic acids are more abundant in terms of concentrations than those present in dietary plants [34]. It was reported that the major flavonoid constituents in fecal water prepared using the samples of 15 human volunteers were quercetin, naringenin, isorhamnetin, formononetin, and hesperetin, with other polyphenolic compounds present at <0.2 μmol/L. In contrast, these fecal water samples were rich in low-molecular-weight phenolic acids, which were present in much higher concentrations than flavonoids [34]. The major components of these phenolic acids were phenylacetic acid, phenylacetic acid, 3-phenylpropanoic acid, 3-(3,4-dihydroxyphenyl)-2-propenoic acid (also called as 3,4-dihydroxycinnamic acid or caffeic acid), 2-(3-hydroxyphenyl)acetic acid, benzoic acid, 3-(4-hydroxyphenyl)propanoic acid, 2-(3,4-dihydroxyphenyl)acetic acid, 2-(4–hydroxyphenyl)acetic acid, 3-(4-hydroxy-3-methoxyphenyl)-2-propenoic acid (also known as 4-hydroxy-3-methoxycinnamic acid or ferulic acid), and 3-(3,4-dihydroxyphenyl)propanoic acid (also known as 3-(3,4-dihydroxyphenyl)propionic acid) [34] (Figure 2). In addition, gut microbiota-derived phenolic acids are more easily absorbed than their parent flavonoids and reach micromolar concentrations in plasma/serum [35].

### 2.1. Generation of Phenolic Acids from Flavonoids by Gut Microbiota

**Figure 3:**
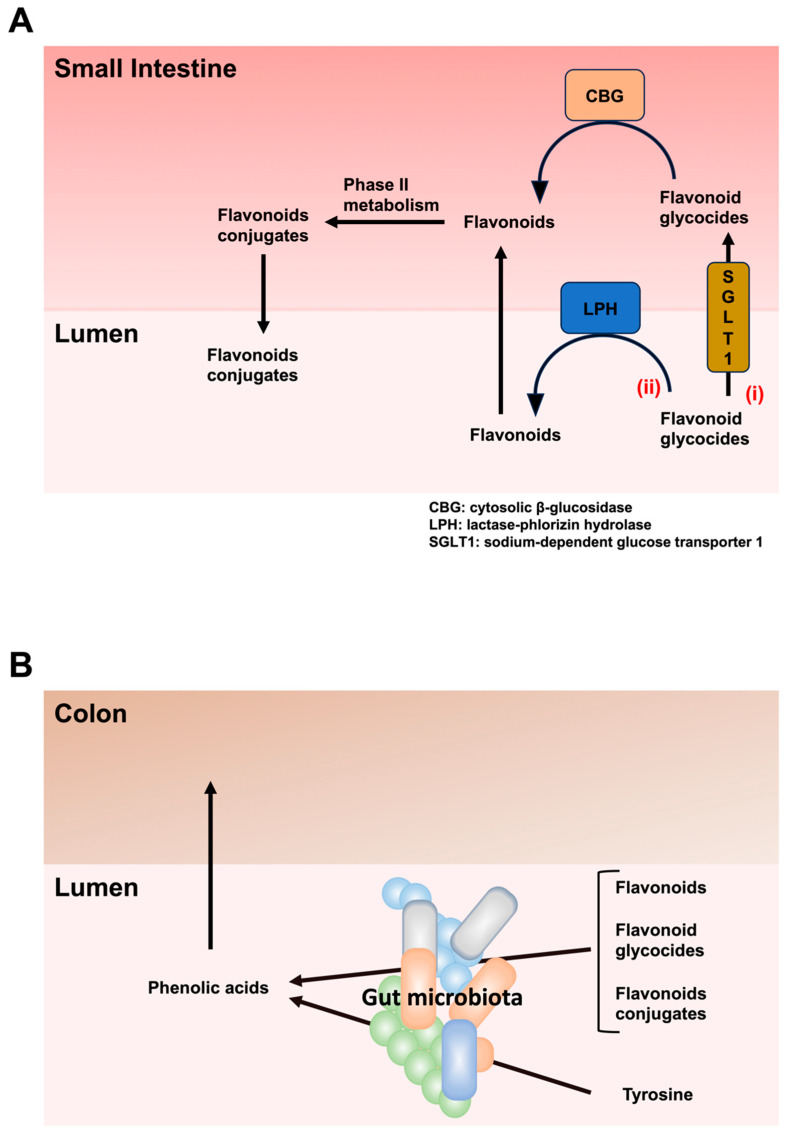
Migration of flavonoids and amino acids to the colon and production of phenolic acids by gut microbiota. (**A**). Flavonoid glycosides can be absorbed into small intestinal epithelial cells via two pathways, (**i**) sodium-dependent glucose transporter 1 (SGLT1) and (**ii**) lactase-phlorizin hydrolase (LPH). (**i**) SGLT1, which is expressed in the intestinal epithelium, transports flavonoid glucosides into small intestinal epithelial cells. After the absorption of flavonoid glycosides by small intestinal epithelial cells, cytosolic β-glucosidase (CBG) hydrolyzes flavonoid glycosides, resulting in the release of flavonoid aglycones. (**ii**) Flavonoid glycosides in the intestinal lumen are hydrolyzed and deglycosylated by LPH, which is primarily expressed at the brush border membrane of small intestinal epithelial cells in the jejunum. After the deglycosylation of flavonoid glycosides by LPH, flavonoid aglycones can pass through the membranes of small intestinal epithelial cells by passive diffusion. Flavonoid aglycones present in the small intestinal epithelial cells undergo phase II metabolism to produce the corresponding conjugated metabolites. Some conjugated flavonoids can be excreted from small intestinal epithelial cells into the gut lumen. (**B**). Flavonoids, flavonoid glycosides that are not absorbed in the small intestine and flavonoid conjugates that are expelled from the small intestine, reach the colon. Bacteria in the gut microbiota metabolize these flavonoids and tyrosine to produce phenolic acids.

Most flavonoids, except catechins, are present in edible plants bound to sugars as β-glycosides. Therefore, most dietary flavonoids cannot be removed by α-monoglucosidase, a major glycolytic enzyme present in pancreatic juice and the small intestine. Hence, the majority of dietary flavonoids are considered poorly absorbed into the circulatory system from the small intestine. Nevertheless, flavonoid monoglycosides can be absorbed into small intestinal epithelial cells via two pathways, viz., a specific glucose transporter and β-glucosidases [36] (Figure 3). Sodium-dependent glucose transporter 1 (SGLT1), which is expressed in the intestinal epithelium and is responsible for transporting hexoses to the systemic circulation [37], transports flavonoid glucosides into small intestinal epithelial cells [38,39,40,41,42,43]. However, it was also reported that several glycosylated forms of flavonoids, such as quercetin, glucoluteolin, apigenin, naringenin, pelargonidin, daidzein, and genistein, are not transported by human SGLT1 expressed in *Xenopus* oocytes [44]. Therefore, it is currently controversial how flavonoid glycosides are directly transported into small intestinal epithelial cells. After flavonoid glycosides are taken into small intestinal epithelial cells, cytosolic β-glucosidase (CBG), which is present within the small intestinal epithelial cells, hydrolyzes flavonoid glycosides, leading to the release of flavonoid aglycones [45,46]. In contrast, flavonoid glycosides in the intestinal lumen are hydrolyzed and deglycosylated by glucosidases present on the brush border membrane of the apical side of small intestinal epithelial cells. This glucosidase is lactase-phlorizin hydrolase (LPH), which is also known as lactase or cytosolic β-glucosidase [47]. LPH is primarily expressed at the brush border membrane of the apical side of small intestinal epithelial cells in the jejunum [45,48,49,50]. After the deglycosylation of flavonoid glycosides, flavonoid aglycones can pass through the membranes of small intestinal epithelial cells by passive diffusion [51,52]. Flavonoid aglycones present in small intestinal epithelial cells undergo phase II metabolism to produce the corresponding conjugated metabolites. The conjugations that produce these conjugated metabolites are glucuronidation by uridine-5′-diphosphate-glucuronosyltransferase, sulfation (also called sulfonation) by sulfotransferase, and methylation by catechol-O-methyltransferase [53]. Although some conjugated flavonoids can be excreted from small intestinal epithelial cells into the gut lumen, the flavonoids absorbed in the small intestinal epithelial cells are transferred to the liver via the portal vein [54,55]. Flavonoids in the liver undergo phases I and II metabolism, and these conjugated flavonoids return to the intestinal lumen via bile [56,57]. Flavonoid glycosides and aglycones that are not absorbed in the small intestine and conjugated flavonoid aglycones that are expelled from small intestinal epithelial cells reach the colon. Bacteria in the gut microbiota have enzymes, such as β-glucosidase, β-glucuronidase, and α-rhamnosidase, that release flavonoid aglycones from flavonoid glycosides [58,59]. Glucoside flavonoids are deglycosylated by β-glucosidase expressed in several bacteria, such as *Bacteroides* spp. [60], *Parabacteroides* spp. [61], *Bifidobacterium* spp. [62,63,64], *Enterococcus* spp. [65], and *Lactobacillus* spp. [63,66]. Moreover, β-glucuronidase expressed in *Escherichia* spp. and *Bacteroides* spp. release flavonoid aglycones from flavonoid glucuronides [67]. In addition, α-rhamnosidase, expressed in *Bifidobacterium* spp., hydrolyzes the terminal rhamnose of various flavonoid glycosides, such as naringin, hesperidin, quercitrin, poncirin, and rutin [68,69,70].

Flavonoid aglycones undergo further metabolism by the gut microbiota, such as the cleavage of A- and C-rings, hydroxylation, dehydroxylation, reduction, decarboxylation, hydrogenation, demethylation, and isomerization [71]. Demethylation is performed by *Eubacterium* spp. [72,73]. Phenolic acids are either absorbed into colon cells via monocarboxylic acid transporters or transported into the blood via paracellular transport through the intercellular space [74]. Furthermore, phenolic acids are present in plasma and can reach various tissues [75,76].

#### 2.1.1. Phenolic Acids from Flavan-3-ols

Flavan-3-ols are abundant in tea, wine, cocoa, and chocolate as well as in vegetables and fruits. Flavan-3-ols have a hydroxyl group at C3 in the C ring of the basic flavonoid skeleton structure (Figure 1).

Epicatechin is converted into 3-(3,4-dihydroxyphenyl)propanoic acid, 3-(3-hydroxyphenyl)propanoic acid (also known as 3-(3-hydroxyphenyl) propionic acid), 5-(3-hydroxyphenyl)pentanoic acid (also known as 5-(3-hydroxyphenyl)valeric acid), and 5-(3,4-dihydroxyphenyl)pentanoic acid (also known as 5-(3,4-dihydroxyphenyl)valeric acid) by human intestinal bacteria in fecal samples [77,78] (Table 1). In addition, flavan-3-ol fractions obtained from grape seeds can be converted into 3-(4-hydroxyphenyl)propanoic acid and 2-(4-hydroxyphenyl)acetic acid by human intestinal bacteria in fecal samples [79]. Moreover, taxifolin is converted into *2*-(3,4-dihydroxyphenyl)acetic acid by *Eubacterium ramulus* [80,81] and *Clostridium orbiscindens* [82]. *Proanthocyanidins are oligomers or polymers of catechin and epicatechin and are metabolized by* the gut microbiota including yeast cells *to produce dihydroxybenzoic acid*, *3,4,5-trihydroxybenzoic acid*, *3-amino-2-hydroxybenzoic acid*, *2-(2-hydroxyphenyl)acetic acid*, *2-(3-hydroxyphenyl)acetic acid*, *2-(4-hydroxyphenyl)acetic acid*, 3-(4-hydroxyphenyl)propanoic acid, *3-(2-hydroxyphenyl)-2-propenoic acid*, *3-(4-hydroxyphenyl)-2-propenoic acid*, and 5-(3-hydroxyphenyl)valeric acid [83,84,85].

#### 2.1.2. Phenolic Acids from Flavanones

Flavanones are present in high levels in grapes and citrus fruits, such as oranges and lemons. Flavanones have a ketone structure at C4 in the C ring of the basic flavonoid skeleton structure (Figure 1).

Naringenin is converted into 3-(4-hydroxyphenyl)propanoic acid by *E. ramulus* [81], *Bifidobacterium longum* R0175, *Lactobacillus rhamnosus* subsp. *rhamnosus* NCTC 10302 [86], *C. orbiscindens* [82], and human intestinal bacteria in fecal samples [87]. Eriodictyol is converted into 3-(3,4-dihydroxyphenyl)propanoic acid by *Clostridium butyricum* [60], *E. ramulus* [81], and *C. orbiscindens* [82]. Hesperetin is converted into 3-(3-hydroxy-4-methoxyphenyl)propanoic acid (also known as 3-(3-hydroxy-4-methoxyphenyl) propionic acid), 3-(3,4-dihydroxyphenyl)propanoic acid, and 3-(3-hydroxyphenyl)propanoic acid by *B. longum* R0175 and is also converted into 3-(3,4-dihydroxyphenyl)propanoic acid and 3-(3-hydroxyphenyl)propanoic acid by *L. rhamnosus* subsp. *rhamnosus* NCTC 10302 [86].

#### 2.1.3. Phenolic Acids from Flavones

Flavones are present in a variety of vegetables and fruits, such as broccoli, carrots, onions, cabbages, and apples. Flavones have a double bond at C2–C3 and a ketone structure at C4 in the C ring of the basic flavonoid skeleton structure (Figure 1).

Luteolin is converted into 3-(3,4-dihydroxyphenyl)propanoic acid by *E. ramulus* [80,81] and *C. orbiscindens* [82]. *In addition*, *homoorientin *(luteolin glucoside) is also converted into 3-(3,4-dihydroxyphenyl)propanoic acid by *Lachnospiraceae* CG-1 [88]. Apigenin is converted into 3-(4-hydroxyphenyl)propanoic acid *by* human intestinal bacteria in fecal samples [89] and *C. orbiscindens* [82]. *Furthermore*, *vitexin *(apigenin 8-C-glucoside) is also converted into 3-(3,4-dihydroxyphenyl)propanoic acid and 3-(4-hydroxyphenyl)propanoic acid by *Lachnospiraceae* CG-1 [88]. *In addition*, *vitexin* is also converted into 3-(4-hydroxyphenyl)propanoic acid by human intestinal bacteria in fecal samples [89].

#### 2.1.4. Phenolic Acids from Flavonols

Flavonols are found in a variety of vegetables and fruits, such as broccoli, tomatoes, onions, grapes, and apples. In addition to these vegetables and fruits, beverages, such as tea and wine, contain flavonols. Flavonols are one of the most analyzed subgroups of flavonoids. They have a double bond at C2–C3, a hydroxyl group at C3, and a ketone structure at C4 in the C ring of the basic flavonoid skeleton structure (Figure 1).

Kaempferol is converted into 2-(3,4-dihydroxyphenyl)acetic acid by *C. orbiscindens* [82] and *E. ramulus* [80,81]. Kaempferol is also converted into 2-(4-hydroxyphenyl)acetic acid by *E. ramulus* [81] and is converted into 3-(4-hydroxyphenyl)-2-propenoic acid (also known as 4-hydroxycinnamic acid or *p*-coumaric acid) and 3-(4-hydroxyphenyl)propanoic acid by human intestinal bacteria in fecal samples [89]. Furthermore, tiliroside (Kaempferol 3-O-β-D-(6″-E-p-coumaroyl)-glucopyranoside) is converted into 3-(4-hydroxyphenyl)-2-propenoic acid and 3-(4-hydroxyphenyl)propanoic acid by human intestinal bacteria in fecal samples [89].

#### 2.1.5. Phenolic Acids from Isoflavones

Isoflavones are abundant in leguminous plants such as soybeans and green beans. Isoflavones have a double bond at C2–C3 and a ketone structure at C4 in the C ring of the basic flavonoid skeleton structure, and the B ring is bound at C3 in the C ring. Genistein can be converted into 3-(4-hydroxyphenyl) propanoic acid by human intestinal bacteria in fecal samples [90].

#### 2.1.6. Phenolic Acids from Anthocyanidins

Anthocyanidins are present in vegetables and fruits such as eggplants, blackcurrants, and blueberries. Anthocyanidins have double bonds at C1–C2 and C3–C4 in the C ring of the basic flavonoid skeleton structure (Figure 1).

*3,4,5*-Trihydroxybenzoic acid is produced from delphinidin and malvidin glycosides by *Bifidobacterium lactis* [63]. Cyanidin 3-glucoside is converted into 3,4-dihydroxybenzoic acid (also known as protocatechuic acid) by human intestinal bacteria in fecal samples, *E. ramulus*, and *Clostridium saccharogumia* [59,91]. Furthermore, *3-(4-hydroxy-3-methoxyphenyl)-2-propenoic acid* is also produced from cyanidin 3-glucoside by human intestinal bacteria from fecal samples [92]. The 4-Hydroxy-3-methoxy benzoic acid (also known as vanillic acid), 4-hydroxybenzoic acid, 2-(4-hydroxyphenyl)acetic acid, and 3-(4-hydroxyphenyl)propanoic acid are produced from cyanidin 3-glucoside by *Bifidobacterium bifidum*, *Bifidobacterium adolescentis*, *Bifidobacterium infantis*, or *Lactobacillus acidophilus* [93]. Malvidin-3-glucoside is converted into 3,4,5-trihydroxybenzoic acid, 4-hydroxy-3,5-dimethoxybenzoic acid (also known as syringic acid), and 3-(4-hydroxyphenyl)-2-propenoic acid, which are produced by human intestinal bacteria in fecal samples [59,92,94].

**Table 1:** Gut bacteria involved in the production of phenolic acids from flavonoids.

Flavonoid Subclasses	Flavonoids	Gut Microbiota-Derived Phenolic Acids	Bacteria Involved in Metabolism	References
**Flavan-3-ols**	Epicatechin	3-(3-Hydroxyphenyl)propanoic acid 3-(3,4-Dihydroxyphenyl)propanoic acid 5-(3-Hydroxyphenyl)pentanoic acid 5-(3,4-Dihydroxyphenyl)pentanoic acid	human intestinal bacteria in fecal samples	[77,78]
	Taxifolin	2-(3,4-Dihydroxyphenyl)acetic acid	*Eubacterium ramulus*	[80,81]
			*Clostridium orbiscindens*	[82]
	Flavan-3-ol fractions	2-(4-Hydroxyphenyl)acetic acid, 3-(4-Hydroxyphenyl)propanoic acid	human intestinal bacteria in fecal samples	[79]
**Flavanones**	Naringenin	3-(4-Hydroxyphenyl)propanoic acid	*Eubacterium ramulus*	[81]
			*Bifidobacterium longum* R0175	[86]
			*Lactobacillus rhamnosus* subsp. *rhamnosus* NCTC 10302	[86]
			*Clostridium orbiscindens*	[82]
			human intestinal bacteria in fecal samples	[87]
	Eriodictyol	3-(3,4-Dihydroxyphenyl)propanoic acid	*Clostridium butyricum*	[60]
			*Eubacterium ramulus*	[81]
			*Clostridium orbiscindens*	[82]
	Hesperetin	3-(3-Hydroxyphenyl)propanoic acid 3-(3,4-Dihydroxyphenyl)propanoic acid 3-(3-Hydroxy-4-methoxyphenyl)propanoic acid	*Bifidobacterium longum R0175*	[86]
		3-(3-Hydroxyphenyl)propanoic acid 3-(3,4-Dihydroxyphenyl)propanoic acid	*Lactobacillus rhamnosus* subsp. *rhamnosus* NCTC 10302	[86]
**Flavones**	Luteolin	3-(3,4-Dihydroxyphenyl)propanoic acid	*Eubacterium ramulus*	[80,81]
			*Clostridium orbiscindens*	[82]
	Homoorientin	3-(3,4-Dihydroxyphenyl)propanoic acid	*Lachnospiraceae* CG-1	[88]
	Apigenin	3-(4-Hydroxyphenyl)propanoic acid	human intestinal bacteria in fecal samples	[89]
			*Clostridium orbiscindens*	[82]
	Vitexin	3-(4-Hydroxyphenyl)propanoic acid	*Lachnospiraceae* CG-1, human intestinal bacteria in fecal samples	[88,89]
		3-(3,4-Dihydroxyphenyl)propanoic acid	*Lachnospiraceae* CG-1	[88]
**Flavonols**	Kaempferol	2-(3,4-Dihydroxyphenyl)acetic acid	*Clostridium orbiscindens*	[82]
			*Eubacterium ramulus*	[80,81]
		2-(4-Hydroxyphenyl)acetic acid	*Eubacterium ramulus*	[81]
		3-(4-Hydroxyphenyl)propanoic acid 3-(4-Hydroxyphenyl)-2-propenoic acid	human intestinal bacteria in fecal samples	[89]
	Tiliroside	3-(4-Hydroxyphenyl)propanoic acid 3-(4-Hydroxyphenyl)-2-propenoic acid	human intestinal bacteria in fecal samples	[89]
**Isoflavones**	Genistein	3-(4-Hydroxyphenyl)propanoic acid	human intestinal bacteria in fecal samples	[90]
**Anthocyanidins**	Delphinidin glycosides Malvidin glycosides	3,4,5-Trihydroxybenzoic acid	*Bifidobacterium lactis*	[63]
	Cyanidin 3-glucoside	3,4-dihydroxybenzoic acid	human intestinal bacteria in fecal samples *Eubacterium ramulus* *Clostridium saccharogumia*	[59,91]
		3-(4-Hydroxy-3-methoxyphenyl)-2-propenoic acid	human intestinal bacteria in fecal samples	[92]
		4-Hydroxybenzoic acid 4-Hydroxy-3-methoxy benzoic acid 2-(4-Hydroxyphenyl)acetic acid 3-(4-hydroxyphenyl)propanoic acid	*Bifidobacterium bifidum* *Bifidobacterium adolescentis* *Bifidobacterium infantis* *Lactobacillus acidophilus*	[93]
	Malvidin-3-glucoside	3,4,5-Trihydroxybenzoic acid 4-Hydroxy-3,5-dimethoxybenzoic acid 3-(4-Hydroxyphenyl)-2-propenoic acid	human intestinal bacteria in fecal samples	[59,92,94]

### 2.2. Generation of Phenolic Acids from Tyrosine by Gut Microbiota

Tyrosine, phenylalanine, and tryptophan are aromatic amino acids that can undergo decarboxylation or transamination. In the decarboxylation pathway, the trace amines tyramine, phenylethylamine, and tryptamine are produced from tyrosine, phenylalanine, and tryptophan, respectively, by the gut microbiota [95,96,97]. Tyramine, phenylethylamine, and tryptamine are ligands for trace amine-associated receptors (TAARs), including TAAR1, which is expressed in a wide variety of tissues [97]. Tyramine is produced by *Enterococcus* spp. [98]. Phenylethylamine can be produced by several species such as *Clostridium* spp., *Blautia* spp., and *Enterocloster* spp. [99]. The production of tryptamine from tryptophan is conducted by *Clostridium* spp. and *Lactobacillus* spp. [100,101].

In contrast, phenolic acids are probably produced from tyrosine by aromatic amino acid aminotransferase [102]. Studies reported that 3-(4-hydroxyphenyl)propanoic acid is produced from tyrosine by *Bacteroides eggerthii*, *Eubacterium hallii*, *Clostridium bartlettii*, and *Clostridium sporogenes* [102,103].

## 3. Effects of Phenolic Acids on Health and Disease

### 3.1. Effects of Phenolic Acids on Infectious Diseases

**Figure 4:**
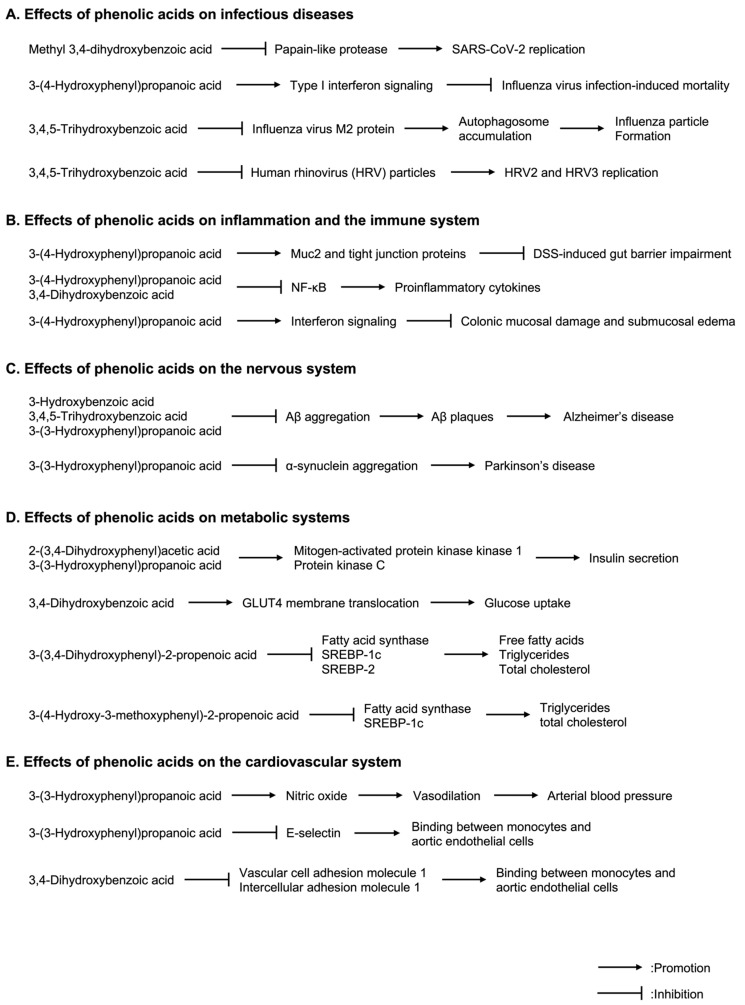
Effects of phenolic acids on health and disease. (**A**) Methyl 3,4-dihydroxybenzoic acid could bind PL^pro^ of SARS-CoV-2, leading to the reduction in SARS-CoV-2 replication. The 3-(4-hydroxyphenyl)propanoic acid suppressed influenza virus infection-induced mortality by enhancing type I interferon signaling. The 3,4,5-trihydroxybenzoic acid inhibited influenza particle formation by inhibiting the production of M2 protein, which promotes the accumulation of autophagosomes and facilitates influenza particle formation. The 3,4,5-trihydroxybenzoic acid also inhibited human rhinovirus 2 (HRV2) and HRV3 replication by interacting with HRV particles. (**B**) The 3-(4-hydroxyphenyl)propanoic acid restored DSS-induced gut barrier impairment by increasing Muc2 and tight junction proteins. The 3-(4-hydroxyphenyl)propanoic acid inhibited the secretion of proinflammatory cytokines by inhibiting NF-κB. The 3,4-dihydroxybenzoic acid decreased the production of proinflammatory cytokines by inhibiting the nuclear translocation of NF-κB. The 3-(4-hydroxyphenyl)propanoic acid also reduced colonic mucosal damage and submucosal edema via type I interferon signaling. (**C**) The 3-hydroxybenzoic acid, 3,4,5-trihydroxybenzoic acid, and 3-(3-hydroxyphenyl)propanoic acid inhibited the aggregation of β-protein (Aβ). Aβ aggregation leads to the formation of Aβ plaques, which are associated with Alzheimer’s disease. The 3-(3-hydroxyphenyl)propanoic acid inhibited the generation of α-synuclein aggregate formation, which is the major pathological characteristics of Parkinson’s disease. (**D**) The 2-(3,4-dihydroxyphenyl)acetic acid and 3-(3-hydroxyphenyl)propanoic acid enhanced insulin secretion by mitogen-activated protein kinase kinase 1 and protein kinase C. The 3,4-dihydroxybenzoic acid exhibited glucose uptake by enhancing GLUT4 membrane translocation. The 3-(3,4-dihydroxyphenyl)-2-propenoic acid decreased the levels of free fatty acids, triglycerides, and total cholesterol by reducing fatty acid synthase, sterol regulatory element-binding protein 1 c (SREBP-1c), a transcription factor for the genes involved in fatty acid and triglyceride synthesis, and SREBP-2, a transcription factor for genes involved in cholesterol synthesis and uptake. The 3-(4-hydroxy-3-methoxyphenyl)-2-propenoic acid decreased the levels of triglycerides, total cholesterol, and HDL and LDL cholesterol by reducing fatty acid synthase and SREBP-1c. (**E**) Effects of phenolic acids on the cardiovascular system. The 3-(3-hydroxyphenyl)propanoic acid decreased arterial blood pressure by nitric oxide-induced vasodilation. The 3-(3-hydroxyphenyl)propanoic acid inhibits the binding between monocytes and aortic endothelial cells by suppressing E-selectin expression in aortic endothelial cells. The 3,4-dihydroxybenzoic acid inhibited the binding between monocytes and aortic endothelial cells by suppressing the expression of vascular cell adhesion molecule 1 and intercellular adhesion molecule 1.

SARS-CoV-2 is related to SARS-CoV-1 and the Middle Eastern respiratory syndrome coronavirus (MERS-CoV) [104] and can cause severe viral pneumonia and even death in the most severe cases [105]. SARS-CoV-2 produces the proteins required for SARS-CoV-2 replication and infection from its own genomic RNA. The SARS-CoV-2 viral genomic RNA encodes structural proteins, which form viral particles, and nonstructural proteins (NSPs), which are essential for replication and other processes, such as proteases and RNA-dependent RNA polymerases. The SARS-CoV-2 protein is cleaved to produce multiple NSPs, such as NSP5 (3C-like protease (3CL^pro^), also known as main protease) and NSP3 (papain-like protease (PL^pro^)). The cleavage occurs at 11 sites by 3CL^pro^ and 3 sites by PL^pro^. PL^pro^ also deconjugates interferon-stimulated gene 15 (ISG15) from interferon regulatory factor (IRF3) and inhibits interferon responses in infected host cells [106]. Furthermore, ISG15 released by PL^pro^ is secreted extracellularly and may induce proinflammatory cytokines, resulting in a cytokine storm [107]. Hence, PL^pro^ plays a crucial role in inhibiting antiviral immunity. Methyl 3,4-dihydroxybenzoic acid could bind PL^pro^ of SARS-CoV-2 and inhibited ISG15 release by PL^pro^. Moreover, methyl 3,4-dihydroxybenzoic acid reduces viral RNA replication and alleviates the cytopathic effects caused by SARS-CoV-2 [108] (Figure 4A) (Table 2).

The 3-(4-Hydroxyphenyl)propanoic acid, produced by *C. orbiscindens*, enhanced the interferon responses induced by interferon-β (IFN-β) and poly (IC). Poly (IC) is a structural analog of double-stranded RNA. The 3-(4-Hydroxyphenyl)propanoic acid is detected in the serum of mice and increases the expression of interferon-γ inducible protein 10 kDa (IP-10), also known as CXCL10, and type I IFN-stimulated genes, such as *2′-5′-oligoadenylate synthetase 2* (*Oas2*) and *myxovirus (influenza virus) resistance 2* (*Mx2*), *in the lungs* of mice [109]. IP-10, *Oas2*, *and Mx2 are associated with antiviral activities in the host* [110,111,112]. Furthermore, 3-(4-hydroxyphenyl)propanoic acid suppressed influenza virus infection-induced mortality by enhancing type I interferon signaling in mice [109] (Figure 4A)*. In addition*, 3-(4-hydroxyphenyl)propanoic acid levels in feces correlated positively with IFN-β in the lungs and negatively with influenza virus infection [113].

The 3,4,5-Trihydroxybenzoic acid inhibited the reduction in the viability of A549 cells (human lung cancer cell line) infected with influenza virus and reduced the viral load of the influenza virus. Influenza virus M2 protein induces autophagosomes and inhibits the fusion of autophagosomes with lysosomes, leading to the accumulation of autophagosomes. The accumulated autophagosomes serve as a source of membrane for influenza particle formation. Inhibition of influenza virus replication by 3,4,5-trihydroxybenzoic acid was due to the inhibition of M2 protein production and the suppression of autophagosome accumulation [114] (Figure 4A). In addition, 3,4,5-trihydroxybenzoic acid also inhibited human rhinovirus 2 (HRV2) and HRV3 replication in HeLa cells (human cervical cancer cell line) by interacting with HRV particles [115] (Figure 4A). Furthermore, 3,4,5-trihydroxybenzoic acid also inhibited the infection of herpes simplex virus-2 (HSV-2) in African green monkey kidney cells by partial inhibition of the adhesion between HSV-2 and host cells [116].

Some phenolic acids were shown to have antiviral activity, such as inhibiting viral replication, enhancing the action of interferon, and increasing the production of proteins involved in antiviral activity, but further elucidation of their mechanism of action is required.

### 3.2. Effects of Phenolic Acids on Inflammation and the Immune System

The 3-(4-hydroxyphenyl)propanoic acid inhibited the secretion of proinflammatory cytokines, including interleukin-1β (IL-1β), IL-6, and tumor necrosis factor-α (TNF-α), from lipopolysaccharide (LPS)-stimulated Caco-2 cells (human colon adenocarcinoma cell line). Furthermore, 3-(4-hydroxyphenyl)propanoic acid reduced the secretion of proinflammatory cytokines, including IL-1β, IL-6, and TNF-α, from the colon and restored colonic edema and destruction of the crypt and luminal surface of the colon in mice with dextran sulfate sodium (DSS)-induced colitis. DSS-induced gut barrier impairment is associated with the reduction in Muc2 and tight junction proteins, such as zonula occludens-1, occludin, claudins, and E-cadherin, and 3-(4-hydroxyphenyl)propanoic acid increased Muc2 and tight junction proteins [117] (Figure 4B). The 3-(4-hydroxyphenyl)propanoic acid inhibited the secretion of proinflammatory cytokines, such as IL-1β and IL-6, and suppressed the formation of foam cells from *RAW264.7 cells* (*mouse macrophage*-like *cell* line) by inhibiting NF-κB [118] (Figure 4B). Foam cells derived from macrophages ingest and accumulate excessive amounts of oxidized LDL cholesterol and release various cytokines to induce a chronic inflammatory response in blood vessels, causing thrombosis and atherosclerosis [119]. The 2-(4-hydroxyphenyl)acetic acid inhibited the production of proinflammatory cytokines, such as IL-1β, IL-6, and TNF-α, and pulmonary edema in the lungs of rats with seawater aspiration-induced lung injury [120]. The 3,4-dihydroxybenzoic acid decreased the production of proinflammatory cytokines, such as TNF-α and IL-1β, in mice by inhibiting the nuclear translocation of NF-κB [121] (Figure 4B) and also decreased the overproduction of cytokines, such as IL-1β and IL-6, in high-fat diet (HFD)-fed mice [122]. The 3-(3,4-dihydroxyphenyl)-2-propenoic acid suppressed IL-1β mRNA expression induced by LPS in RAW 264.7 [123]. In addition, 4-hydroxybenzoic acid inhibited the IL-1β secretion from LPS-treated human THP-1 cells [124].

The 3-(4-hydroxyphenyl)propanoic acid increased the number of CD8^+^ T cells expressing IFN-γ and the expression of the early activation marker CD25 by TCR stimulation with CD3/CD28-coated beads and enhanced T-cell activation. It also induced dendritic cell maturation by enhancing the surface expression of the co-stimulatory molecule CD80 or CD86 on dendritic cells treated with LPS [125]. The 3-(4-hydroxyphenyl)propanoic acid also reduced colonic mucosal damage and submucosal edema in HFD- and DSS-fed mice via type I interferon signaling (Figure 4B). It also protected against death induced by LPS administration in mice [126]. The 5-(3,4,5-trihydroxyphenyl)pentanoic acid (also known as 5-(3,4,5-trihydroxyphenyl)valeric acid) reduced ATP levels in mouse CD4^+^ cells. ATP levels in CD4^+^ cells indicate the activity of CD4^+^ cells [127].

Several phenolic acids were reported to have immunosuppressive effects, such as suppressing proinflammatory cytokines. Among them, 3-(4-hydroxyphenyl)propanoic acid was reported to not only suppress cytokines but also activate immune cells, such as activating T cells and inducing dendritic cells. It is very important to maintain a proper balance between immune activation and suppression, and if this balance is disturbed, it can lead to immune system diseases such as autoimmune diseases and immunodeficiencies. Therefore, 3-(4-hydroxyphenyl)propanoic acid may be able to play an important role in regulating this balance in the immune system.

### 3.3. Effects of Phenolic Acids on the Nervous System

Flavonoids and phenolic acids can pass through the blood–brain barrier (BBB) and influence the central nervous system [11,128,129,130]. It was reported that 3-(4-hydroxyphenyl) propanoic acid, 3-(3-hydroxyphenyl) propanoic acid, 2-(3,4-dihydroxyphenyl)acetic acid, and 2-(3-hydroxyphenyl)acetic acid exerted protective effects against cell death in human SH-SY5Y neuroblastoma cells treated with 3-morpholinosydnonimine, a donor of superoxide, nitric oxide, and peroxynitrite [131].

The 3-(3-hydroxyphenyl)propanoic acid and 3-hydroxybenzoic acid inhibited the aggregation of β-protein (Aβ) in vitro [130]. The 3,4-dihydroxybenzoic acid reduced Aβ levels in the hippocampus and cerebral cortex of amyloid precursor protein (APP)/presenilin-1 (PS1) transgenic Alzheimer’s disease (AD) model mice [132]. It also reduced ischemia-induced hippocampal neuronal death and BBB disruption and improved cognitive function after ischemia in rats [133]. The 3,4,5-trihydroxybenzoic acid improved cognitive functions and reduced Aβ plaques in APP/PS1 transgenic AD model mice by inhibiting Aβ aggregation [134] (Figure 4C). Moreover, 3,4,5-trihydroxybenzoic acid and 4-hydroxy-3-methoxy benzoic acid recovered myelination from lysophosphatidylcholine (LPC)-induced demyelination in mice [135].

The 3-(3-dydroxyphenyl)propanoic acid inhibited the generation of α-synuclein aggregate formation in vitro and in cells [136,137] (Figure 4C). Aggregation and deposition of α-synuclein are the major pathological characteristics of Parkinson’s disease [138]. The 4-hydroxy-3,5-dimethoxybenzoic acid reduced dyscoordination in model mice with Parkinson’s disease treated with 1-methyl-4-phenyl-1,2,3,6-tetrahydropyridine (MPTP) and probenecid [139]. The 4-hydroxybenzoic acid protected cerebellar granule neurons from damage induced by glutamate [140].

Some phenolic acids were reported to have the effect of lowering β protein levels and inhibiting the aggregation of β protein and α-synuclein. Further research into these phenolic acids is expected to lead to the development of new treatments and drugs for neurodegenerative diseases such as AD and PD.

### 3.4. Effects of Phenolic Acids on Metabolic Systems

The 2-(3,4-dihydroxyphenyl)acetic acid and 3-(3-hydroxyphenyl)propanoic acid enhanced glucose-stimulated insulin secretion in isolated rat islets by mitogen-activated protein kinase kinase 1 and protein kinase C [141] (Figure 4D). The 3-(3-hydroxyphenyl)propanoic acid and 3-(4-hydroxyphenyl)propanoic acid improved the levels of triglycerides, total cholesterol, HDL and LDL cholesterol, and free fatty acids in the serum of HFD-fed mice [142]. The 3-(4-hydroxyphenyl)propanoic acid reduced subcutaneous and epididymal fat and also reduced the levels of blood glucose [126].

The 3,4-dihydroxybenzoic acid decreased hemoglobin A1C (HbA1c) and blood glucose levels in rodents [143,144,145,146,147]. It also decreased body weight and blood glucose levels in HFD-fed mice [122]. Moreover, 3,4-dihydroxybenzoic acid also exhibited insulin-like activities, such as glucose uptake by enhancing GLUT4 membrane translocation in human adipocytes [148] (Figure 4D). It also decreased the levels of HDL and LDL cholesterol, triglycerides, glycohemoglobin, and advanced glycation end products in rats [147]. The 3,4,5-trihydroxybenzoic acid reduced HbA1c and blood glucose levels in rats with streptozotocin-induced diabetes [149] and mice fed with HFD [150].

The 3-(4-hydroxyphenyl)-2-propenoic acid reduced HbA1c and blood glucose levels in rats with streptozotocin-induced diabetes [149]. The 3-(3,4-dihydroxyphenyl)-2-propenoic acid decreased the levels of free fatty acids, triglycerides, and total cholesterol in HFD-fed mice by reducing fatty acid synthase, sterol regulatory element-binding protein 1 c (SREBP-1c), a transcription factor for the genes involved in fatty acid and triglyceride synthesis, and SREBP-2, a transcription factor for genes involved in cholesterol synthesis and uptake [151] (Figure 4D). The 3-(4-hydroxy-3-methoxyphenyl)-2-propenoic acid decreased blood glucose levels in HFD-fed mice [152]. It also decreased the levels of triglycerides, total cholesterol, and HDL and LDL cholesterol in HFD-fed rodents by fatty acid synthase and SREBP-1c [153,154] (Figure 4D).

It was reported that phenolic acids have effects such as improving blood sugar, triglyceride, and HDL cholesterol levels and reducing subcutaneous fat and therefore they may be linked to the improvement of metabolic syndrome and obesity.

### 3.5. Effects of Phenolic Acids on the Cardiovascular System

The 3-(3-hydroxyphenyl) propanoic acid, 2-(3-hydroxyphenyl)acetic acid, and 2-(3,4-dihydroxyphenyl)acetic acid decreased arterial blood pressure in rats [155,156,157]. This decrease in blood pressure by 3-(3-hydroxyphenyl)propanoic acid was caused by nitric oxide-induced vasodilation [155,157] (Figure 4E). Furthermore, 3-(3-hydroxyphenyl)propanoic acid inhibits the binding between monocytes and aortic endothelial cells by suppressing E-selectin expression in aortic endothelial cells [158] (Figure 4E). Because the binding between monocytes and vascular endothelial cells is the initiating step of atherogenesis, 3-(3-hydroxyphenyl)propanoic acid may inhibit atherogenesis.

The 3,4-dihydroxybenzoic acid inhibits cardiac hypertrophy and improves cardiac functions, such as fractional shortening and ejection fraction, in mice with isoproterenol-treated heart failure [159]. Moreover, 3,4-dihydroxybenzoic acid reduces the accumulation of cholesterol in the aortic sinus and improves atherosclerotic lesions in the aortic sinus in mice [160]. It also inhibits the binding between monocytes and aortic endothelial cells by suppressing the expression of vascular cell adhesion molecule 1 and intercellular adhesion molecule 1 in aortic endothelial cells [160] (Figure 4E).

The 5-(3-hydroxyphenyl)pentanoic acid, 5-(3,5-dihydroxyphenyl)pentanoic acid (also known as 5-(3,5-dihydroxyphenyl)valeric acid), and 5-(3,4,5-trihydroxyphenyl)pentanoic acid inhibit the angiotensin I-converting enzyme, which converts angiotensin I into angiotensin II. Angiotensin II is a physiologically active peptide that increases blood pressure [161].

Phenolic acids not only improve cardiac function by lowering blood pressure, suppressing cardiac hypertrophy, and improving atherosclerotic lesions in the aortic sinus but also were reported to inhibit the binding of monocytes to aortic endothelial cells, which is an early stage of atherosclerosis, and it is expected that phenolic acids may also have a preventive effect against atherosclerosis.

**Table 2:** Effects of phenolic acids on health and disease.

	Phenolic Acids	Biological Effects	References
**Infectious** **diseases**	Methyl 3,4-Dihydroxybenzoic acid	Reduction in viral RNA replication of SARS-CoV-2 Inhibition of ISG15 release by binding to papain-like protease	[108]
	3,4,5-Trihydroxybenzoic acid	Reduction in the viral load of the influenza virus	[114]
		Inhibition of human rhinovirus	[115]
		Inhibition of the infection of herpes simplex virus-2	[116]
	4-Hydroxy-3-methylbenzoic acid	Enhancement of interferon responses	[109]
	3-(4-Hydroxyphenyl)propanoic acid	Enhancement of interferon responses, prevention of mortality due to influenza	[109]
**Immune** **system**	4-Hydroxybenzoic acid	Inhibition of the secretion of proinflammatory cytokines	[124]
	3,4-Dihydroxybenzoic acid	Inhibition of the secretion of proinflammatory cytokines	[121,122]
	2-(4-Hydroxyphenyl)acetic acid	Inhibition of the secretion of proinflammatory cytokines and pulmonary edema in the lungs	[120]
	3-(4-Hydroxyphenyl)propanoic acid	Inhibition of the secretion of proinflammatory cytokines	[117,118]
		Enhancement of T-cell activation, induction of dendritic cell maturation	[125]
		Reduction in colonic damage and edema	[117,126]
	3-(3,4-Dihydroxyphenyl)-2-propenoic acid	Inhibition of the secretion of proinflammatory cytokines	[123]
	5-(3,4,5-Trihydroxyphenyl)pentanoic acid	Reduction in the activity of CD^4+^ cells	[127]
**Nervous** **system**	3-Hydroxybenzoic acid	Inhibition of the aggregation of β-protein	[130]
	4-Hydroxybenzoic acid	Protective effects against glutamate-induced neuronal damage	[140]
	3,4-Dihydroxybenzoic acid	Reduction in β-protein levels	[132]
		Neuronal death, BBB disruption, improvement of cognitive function	[133]
	3,4,5-Ttrihydroxybenzoic acid	Improvement of cognitive function, reduction in β-protein plaques	[134]
		Recovery of myelination	[135]
	4-Hydroxy-3-methoxy benzoic acid	Recovery of myelination	[135]
	4-Hydroxy-3,5-dimethoxybenzoic acid	Reduction in dyscoordination in model mice with Parkinson’s disease	[139]
	2-(3-Hydroxyphenyl)acetic acid	Protective effects against cell death in human SH-SY5Y neuroblastoma cells	[131]
	2-(3,4-Dihydroxyphenyl)acetic acid	Protective effects against cell death in human SH-SY5Y neuroblastoma cells	[131]
	3-(3-Hydroxyphenyl)propanoic acid	Protective effects against cell death in human SH-SY5Y neuroblastoma cells	[131]
		Inhibition of the aggregation of β-protein	[130]
		Inhibition of α-synuclein aggregation	[136,137]
	3-(4-Hydroxyphenyl)propanoic acid	Protective effects against cell death in human SH-SY5Y neuroblastoma cells	[131]
**Metabolic** **systems**	3,4-Dihydroxybenzoic acid	Reduction in hemoglobin A1C, blood glucose levels, and body weight	[122,143,144,145,146,147]
		Reduction in the levels of HDL and LDL cholesterol, triglycerides, glycohemoglobin, and advanced glycation end products	[147]
	3,4,5-Trihydroxybenzoic acid	Reduction in hemoglobin A1C and blood glucose levels	[149,150]
	2-(3,4-Dihydroxyphenyl)acetic acid	Enhancement of glucose-stimulated insulin secretion	[141]
	3-(3-Hydroxyphenyl)propanoic acid	Enhancement of glucose-stimulated insulin secretion	[141]
		Improvement of the levels of triglycerides, total cholesterol, HDL and LDL cholesterol, and free fatty acids	[142]
	3-(4-Hydroxyphenyl)propanoic acid	Improvement of the levels of triglycerides, total cholesterol, HDL and LDL cholesterol, and free fatty acids	[142]
		Reduction in subcutaneous and epididymal fat and the levels of blood glucose	[126]
	3-(4-Hydroxyphenyl)-2-propenoic acid	Reduction in hemoglobin A1C and blood glucose levels	[149]
	3-(3,4-Dihydroxyphenyl)-2-propenoic acid	Reduction in the levels of blood glucose, free fatty acids, triglycerides, and total cholesterol	[151]
	3-(4-Hydroxy-3-methoxyphenyl)-2-propenoic acid	Reduction in the levels of glucose, triglycerides, total cholesterol, and HDL and LDL cholesterol	[152,153,154]
**Cardiovascular system**	3,4-Dihydroxybenzoic acid	Inhibition of cardiac hypertrophy and improvement of cardiac functions	[159]
		Reduction in the accumulation of cholesterol in the aortic sinus Improvement of atherosclerotic lesions in the aortic sinus	[160]
		Inhibition of the binding between monocytes and aortic endothelial cells	[160]
	2-(3-Hydroxyphenyl)acetic acid	Reduction in arterial blood pressure	[157]
	2-(3,4-Dihydroxyphenyl)acetic acid	Reduction in arterial blood pressure	[156]
	3-(3-Hydroxyphenyl)propanoic acid	Reduction in arterial blood pressure	[155,156]
		Inhibition in the binding between monocytes and aortic endothelial cells	[158]
	5-(3-Hydroxyphenyl)pentanoic acid	Inhibition of the angiotensin I-converting enzyme	[161]
	5-(3,5-Dihydroxyphenyl)pentanoic acid	Inhibition of the angiotensin I-converting enzyme	[161]
	5-(3,4,5-Trihydroxyphenyl)pentanoic acid	Inhibition of the angiotensin I-converting enzyme	[161]

## 4. Concluding Remarks

Diet exerts a significant impact not only on human health but also on gut bacteria. Fruits, vegetables, and grains are rich in flavonoids, whereas meat, fish, soybeans, and cheese are rich in amino acids. Therefore, diverse flavonoids and amino acids are present in the foods and beverages we consume daily. After these flavonoids and amino acids in the diet reach the intestine, metabolic activity by the intestinal bacteria of the gut microbiota occurs, producing a variety of metabolites, including phenolic acids. Phenolic acids pproduced by the gut microflora were shown to be present in the brain as well as in the systemic circulation. Therefore, gut microbiota-derived phenolic acids can exert a variety of effects on the entire body. Therefore, phenolic acids produced by the gut microbiota from dietary flavonoids and amino acids can be considered postbiotics, such as short-chain fatty acids and secondary bile acids. These phenolic acids have antiviral effects against several viruses, such as SARS-CoV-2, influenza, HRVs, and HSV-2, and can influence the immune, nervous, metabolic, and cardiovascular systems. Furthermore, recent studies demonstrated that fecal microbiota transplantation influences the production of phenolic acids that may be involved in health and diseases [162,163]. Fecal microbiota transplantation from normal mice to DSS-induced colitis mice induces 2-hydroxy-3-(4-hydroxyphenyl)propionic acid (also known as hydroxyphenyllactic acid) in feces [163]. The 2-hydroxy-3-(4-hydroxyphenyl)propionic acid can reduce excessive reactive oxygen species in neutrophils and protect organs from sepsis [164]. Compared to fecal microbiota transplants from low-fat diet mice, normal recipient mice receiving fecal microbiota transplants from high-fat diet (HFD) mice had increased levels of 3-(3-hydroxyphenyl)propanoic acid and 3-(4-hydroxyphenyl)propanoic acid in plasma and cecum were elevated. Furthermore, daily intra-peritoneal injection of 3-(3-hydroxyphenyl)propanoic acid to normal mice increased the expression of genes involved in dopaminergic reaction (dopamine receptor 1 and 2), opioid reaction (κ-opioid receptor and μ-opioid receptor), and preprodynorphin in the brain. These genes are related to a food reward, leading to obesity [162]. These findings indicate that fecal microbiota transplantation leads to beneficial effects on diseases. Although most of these studies were on the effects of phenolic acids in vitro or in animals, phenolic acids were shown to be potentially useful in the control or amelioration of various diseases, leading to health. Hence, it is necessary to elucidate the detailed mechanisms involved in the synthesis, bioavailability, and physiological functions of phenolic acids derived from flavonoids and amino acids by the gut microbiota, which would help in developing novel therapeutic strategies and preventive medicine for various diseases.

Further studies are required on the metabolites of the gut microbiota to elucidate their benefits in human health and diseases.
